# Sudden losses and sudden gains during a DBT-PTSD treatment for posttraumatic stress disorder following childhood sexual abuse

**DOI:** 10.3402/ejpt.v5.24470

**Published:** 2014-09-17

**Authors:** Antje Krüger, Thomas Ehring, Kathlen Priebe, Anne S. Dyer, Regina Steil, Martin Bohus

**Affiliations:** 1 Institute of Psychology, University of Münster, Münster, Germany; 2 Department of Psychosomatic Medicine and Psychotherapy, Central Institute of Mental Health, Medical Faculty Mannheim/Heidelberg University, Mannheim, Germany; 3 Department of Psychology and Psychotherapy, School of Social Science, University of Mannheim, Mannheim, Germany; 4 Department of Psychology and Sports Sciences, Institute of Psychology, Johann Wolfgang Goethe University, Frankfurt/Main, Germany

**Keywords:** Posttraumatic stress disorder, symptom worsening, sudden changes, exposure-based treatment

## Abstract

**Background:**

Exposure-based treatment approaches are first-line interventions for patients suffering from posttraumatic stress disorder (PTSD). However, the dissemination of exposure-based treatments for PTSD is challenging, as a large proportion of clinicians report being concerned about symptoms worsening as a result of this type of intervention and are therefore reluctant to offer it to patients with PTSD. However, there is only little empirical evidence to date on the pattern of symptom worsening during exposure-based treatment for PTSD.

**Objective:**

The goal of the present study was to explore the frequency of sudden losses and sudden gains in the course of an exposure-based treatment programme for female patients suffering from PTSD related to childhood sexual abuse who also show severe comorbidity. In addition, the relationship between sudden changes and treatment outcome was examined.

**Methods:**

Female participants (*N*=74) were randomised to either a 12-week residential DBT-PTSD programme or a treatment-as-usual wait list. The pattern of symptom change was assessed via weekly assessments using the Posttraumatic Diagnostic Scale (PDS). Sudden changes were computed as suggested by the literature on sudden gains.

**Results:**

During treatment, only one participant (3%) experienced a sudden loss, whereas 25% of participants experienced sudden gains. In the waiting condition, 8% of the participants experienced sudden losses and 5% experienced sudden gains during the same time period. No symptom worsening was observed in response to exposure sessions. However, sudden gains occurred during exposure and non-exposure treatment weeks. Patients with sudden gains showed better treatment outcome in the post-treatment and follow-up assessments.

**Conclusions:**

Exposure-based treatment did not lead to PTSD symptom worsening in the study sample. Results show that sudden gains occur frequently during PTSD treatment and have a prognostic value for treatment outcome.

Survivors of childhood sexual abuse (CSA) show a high risk for the development of posttraumatic stress disorder (PTSD) and other co-occurring problems (Fergusson, McLeod, & Horwood, 2013) with an odds ratio (OR) of 7.3 for PTSD, 8.0 for substance abuse and 7.6 for borderline personality disorder (BPD) in a female sample of CSA survivors (Cutajar et al., 2010). In addition, co-occurring problems that do not necessarily meet criteria for a separate disorder, such as low self-esteem, interpersonal problems, suicidal ideation or non-suicidal self-injury (NSSI), are frequently presented in this group (Zlotnick et al., 2003).

The efficacy of psychological treatments for PTSD has extensively been examined. Conclusions from recent meta-analyses and systematic reviews concur that trauma-focused cognitive-behavioural therapy and eye movement desensitization and reprocessing (EMDR) consistently show high effect sizes and are therefore regarded as first-line treatments (Bisson et al., 2007; Bradley, Greene, Russ, Dutra, & Westen, 2005; Cloitre, 2009). Exposure-based interventions are especially recommended (Foa, Keane, Friedman, & Cohen, 2009). Reassuringly, research focusing specifically on PTSD following CSA shows that trauma-focused treatment approaches including exposure interventions are also efficacious in this group of trauma survivors (Cloitre et al., 2010; Taylor & Harvey, 2010).

In spite of the well-proven efficacy of trauma-focused treatments for PTSD, some therapists question the safety of exposure-based treatment for PTSD patients and are reluctant to use these interventions (Becker, Zayfert, & Anderson, 2004; Cahill, Foa, Hembree, Marshall, & Nacash, 2006; Jaycox & Foa, 1996) and fear that exposure-based treatment may lead to symptom worsening or exacerbation of comorbid symptoms (Cahill et al., 2006; Feeny, Hembree, & Zoellner, 2003). Because of the complex symptomatology often found in patients with PTSD following CSA (Cloitre et al., 2009), this concern is especially salient in the treatment of this population.

A number of authors have emphasized that there is currently no evidence to suggest that exposure-based treatment is related to symptom worsening on a large scale (e.g., Cahill et al., 2006; Van Minnen, Harned, Zoellner, & Mills, 2012). Only recently, an article on symptom worsening in a large sample of PTSD patients (*n*=361) reported an overall improvement on PTSD and showed that worsening of PTSD symptoms was virtually non-existent from pre- to post-treatment assessments (Jayawickreme et al., 2014). Reliable worsening in depressive symptoms was observed in 2% of the participants. However, it remains unclear whether sudden worsening during treatment occurred following specific treatment sessions. In earlier publications, symptom worsening in exposure-based treatment has been reported in few studies (Pitman et al., 1991; Tarrier et al., 1999). Important methodological problems regarding the definition of symptom worsening in these studies have been highlighted. For example, Devilly and Foa (2001) argue that symptom worsening should be defined as a reliable increase in symptoms that goes beyond normal symptom fluctuation. Studies following this definition found that although single patients did show reliable symptom worsening during treatment, these patients nevertheless benefitted from the treatment to a comparable degree as patients without worsening as evidenced by their post-treatment scores (Foa, Zoellner, Feeny, Hembree, & Alvarez-Conrad, 2002; Hembree et al., 2003).

To our knowledge, only a few randomised-controlled trials to date have assessed symptom worsening in patients with PTSD following CSA (Chard, 2005; Cloitre, Koenen, Cohen, & Han, 2002; Cloitre et al., 2010). In one study, none of the patients showed an increase in PTSD symptoms at post-treatment, assessed with the Clinician-Administered PTSD Scale (CAPS; Blake et al., 2000) at pre- and post-treatment (Chard, 2005). Cloitre et al. (2002) reported an increase in PTSD symptoms measured with the CAPS interview in 5% of the patients in the treatment group, compared to 25% in the waiting list. In a later study, Cloitre et al. (2010) compared three different phase-based treatment protocols. Symptom worsening here was defined as an increase of seven or more points on the CAPS from pre- to post-treatment. The frequency of symptom worsening between these two assessments ranged from 3.6 to 15% in the different study arms, with no significant differences between them. The amount of symptom worsening *during* treatment was not reported. Therefore, it remains unclear at exactly which point in treatment the exacerbation of symptoms occurred and whether there were any instances of symptom worsening during treatment that normalized until the end of the intervention. From a clinical point of view, it appears highly relevant to not only study symptom worsening from pre- to post-treatment but also investigate acute symptom worsening (sudden losses) during the course of treatment. About 20% of patients have been shown to drop out of PTSD treatment (Imel, Laska, Jakupcak, & Simpson, 2013). If sudden losses are found to frequently occur during PTSD treatment, this may be one of the reasons for the relatively high attrition rates. In addition, detailed information on the pattern of sudden symptom worsening during treatment is necessary to decide whether clinicians’ fear of symptom exacerbation is justified or not. Finally, knowledge about the exact timing of symptom worsening in the course of PTSD treatment may help to identify critical phases.

On the other hand, investigating sudden gains, and their relationship to treatment outcome appears equally important but under-researched in PTSD. Research into this phenomenon has originated in the literature on the treatment of depression (Tang & DeRubeis, 1999). There is now extensive evidence showing that in patients with depression and anxiety disorders sudden gains during treatment are associated with better treatment outcome (Aderka, Nickerson, Boe, & Hofmann, 2012; Kelly, Roberts, & Ciesla, 2005) and lower risk of relapse (Tang, DeRubeis, Hollon, Amsterdam, & Shelton, 2007).

In the PTSD literature, sudden gains have been reported in only four studies to date. Jun, Zoellner, and Feeny (2013) compared sudden gains in patients with chronic PTSD treated with prolonged exposure (PE) or pharmacotherapy. They found sudden gains in 42% of patients in the PE condition, and in 31% of patients in the pharmacotherapy condition. Aderka, Appelbaum-Namdar, Shafran, and Gilboa-Schechtman (2011) found sudden gains in 49% of their paediatric sample, and sudden gains predicted better outcomes at post-treatment and follow-up. A comparable number of sudden gains (52%) as well as a relationship with post-treatment outcome was found by Doane, Feeny, and Zoellner (2010); however, no information on the stability of this effect to follow-up is provided. Kelly, Rizvi, Monson, and Resick (2009) observed sudden gains in fewer patients (39%). These sudden gains were associated with better treatment outcome at post-treatment for PTSD and depression. However, at follow-up assessment the sudden gains group only remained to show superior outcome for depression, but not anymore for PTSD. In summary, sudden gains seem to occur in PTSD treatments and to be related to better treatment outcome. However, it remains unclear whether this is also the case when looking at long-term treatment effects. In addition, no study to date has simultaneously examined sudden losses in addition to sudden gains.

The objective of the present study was to examine sudden changes in PTSD symptoms during the course of an exposure-based PTSD treatment (DBT-PTSD) in comparison to a treatment-as-usual waiting list (TAU-WL). The comparison between TAU-WL and DBT-PTSD helps to understand whether sudden changes are specific to treatment or whether these are natural symptom fluctuations. As there is no evidence to date whether sudden gains occur more or less frequently in response to exposure sessions than in response to other interventions, a second goal of the present study was to explore a possible relationship between exposure interventions and sudden gains. The treatment group received dialectical behaviour therapy for PTSD in a residential setting (DBT-PTSD; Bohus et al., 2013). The treatment protocol addressed the specific needs of patients suffering from PTSD following CSA and borderline-typical co-occurring problems.

The following hypotheses were tested: 1) As there is no literature on sudden losses during PTSD treatment to date, we explored whether participants in the treatment group differed from those in the waiting list condition with regard to the number of sudden losses experienced. 2) In the DBT-PTSD group, sudden gains occur more frequently than in the TAU-WL. 3) On an exploratory level, it was tested whether sudden gains are significantly more likely following sessions including exposure than following sessions without exposure. 4) Patients with sudden gains show a better treatment outcome at post-treatment and follow-up in comparison to patients without sudden gains and in comparison to patients with sudden losses. Further exploratory analyses tested whether sudden changes were associated with pre-treatment PTSD symptom severity, general psychopathology, number of diagnoses, number of borderline-criteria, and NSSI pre-treatment and during treatment.

## Methods

### Participants

**Fig. 1:**
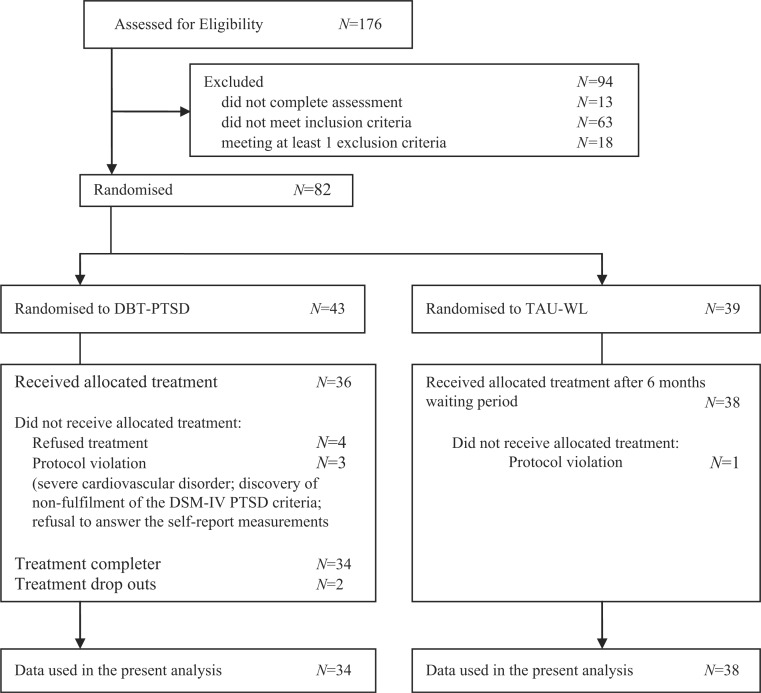
Flow chart.

Participants included 74 women who were randomly assigned to an active treatment (DBT-PTSD; *n =* 36) or to a TAU-WL (*n*=38) (Bohus et al., 2013). Inclusion criteria included: aged 17–65 years, a DSM-IV diagnosis of PTSD related to CSA and at least one of the following additional diagnoses: eating disorder, current major depression, current substance abuse, or meeting ≥4 DSM-IV criteria for BPD. Exclusion criteria were a lifetime diagnosis of schizophrenia, current substance dependence, body mass index <16.5, intellectual disability, and medical conditions contradicting the exposure protocol (e.g., severe cardiovascular disorder). See Fig. 1 for more details about the patient flow.

Hypotheses (1) and (2) were tested using the whole sample. All remaining analyses were conducted with the DBT-PTSD subgroup only.

### Measures

PTSD symptoms were assessed by the *Clinician-Administered PTSD Scale* (CAPS*;* Blake et al., 2000), a structured interview according to the DSM-IV (American Psychiatric Association, 2000) and the *Posttraumatic Diagnostic Scale* (PDS; Foa, Cashman, Jaycox, & Perry, 1997), a self-report measure of PTSD symptoms. The PDS was not only used as an outcome measure but also as a process measure in the present study.

Axis I and II disorders were diagnosed by the *Structured Clinical Interview for DSM-IV Axis I Disorders* (SCID-I; First, Spitzer, Gibbon, Williams, & Benjamin, 1997) and the *International Personality Disorder Examination* (IPDE; Loranger et al., 1994). NSSI and suicide attempts pre-treatment were assessed using the *Severe Behaviour Dyscontrol Interview* (SBD-I; Bohus & Borgmann, 2012), which is based on the Suicide Attempt Self-Injury Interview (SASII; Linehan, Comtois, Brown, Heard, & Wagner, 2006). During treatment, NSSI was assessed daily using a DBT-specific self-report diary card. At pre-post-treatment and follow-up, self-report measures were used to assess depressive symptoms (*Beck Depression Inventory;* BDI-II; Beck, Steer, & Brown, 1996), global psychopathology (*Symptom Checklist-90-Revised;* SCL-90-R; Derogatis, 1992) and borderline symptoms (*Borderline Symptom List;* BSL-23; Bohus et al., 2009). Results on these self-report measures at post-treatment and follow-up assessment are reported elsewhere (see Bohus et al., 2013) as they are beyond the scope of this article.

### Procedure

Participants were assessed at randomisation (t1), as well as 12 weeks (t2), 18 weeks (t3) and 24 weeks (t4) later, which corresponded for the treatment group to the time points of admission (t1), discharge (t2), 6 weeks follow-up (t3) and 12 weeks follow-up (t4). Additionally, PTSD symptoms were assessed weekly during the whole assessment period using the PDS.

### Treatment protocol

DBT-PTSD is a modular multi-component treatment protocol that is based on three treatment phases. In Phase 1 (Weeks 1–4), patients are taught to identify their individual avoidance strategies on a cognitive, emotional and behavioural level. With the help of individualised behavioural analyses, they learn to use specific DBT skills to control their behaviour. There are no exposure interventions in this treatment phase. Phase 2 (Weeks 5–10) focuses on trauma-specific cognitive and exposure-based interventions. Exposure started on average in Week 4 and continued until discharge. However, the beginning and the duration of exposure interventions were individualised based on clinical decisions on the background of the DBT-PTSD treatment hierarchy. Treatment Phase 2 therefore includes treatment weeks with and without exposure interventions. Phase 3 (Weeks 11 and 12) aims at improving radical acceptance of trauma-related and biographical facts.

In the TAU-WL condition participants received 6 months of treatment of their choice. With the exception of DBT-PTSD, the kind of treatment or the treatment setting (i.e., residential or outpatient setting) during this time was not controlled. After 6 months they were admitted to the residential setting and offered DBT-PTSD treatment. However, the delayed DBT-PTSD treatment was not part of the study design. Therefore, during and post-treatment no systematic data collection took place, which is why this phase was not included in the data analysis.

More details of the DBT-PTSD treatment protocol are described elsewhere (Bohus et al., 2013; Steil, Dyer, Priebe, Kleindienst, & Bohus, 2011).

### Data analyses

#### Symptom change calculation

In earlier studies, symptom worsening has mainly been investigated by comparing pre- and post-measurements, but has not been assessed during treatment in terms of acute worsening. In order for the criteria for sudden gains and sudden losses to be consistent, we adopted the formula to calculate sudden gains for the measurements of sudden losses. Sudden change between sessions *N* and *N*+1 was computed following the definition suggested by Tang and DeRubeis (1999). On a general level, symptom change has to be meaningful and not due to the measurement error of the assessment, which is defined as a change that is 1) large in absolute terms, 2) represents at least 25% symptom change from the pre-change level of symptoms, and 3) represents a stable change that is larger than normal symptom fluctuation before and after the change (Lutz et al., 2013; Tang & DeRubeis, 1999). In the current study, these criteria were operationalized as follows:

1. A change is required that is not due to the standard error of measurement (SEM). The SEM at any point of time on the PDS is calculated as described by Devilly and Foa (2001) using the 2- to 3-week test–retest reliability (*r=*0.83) and standard deviation (SD*=*10.54) for the sum score originally reported for the PSS-SR (Foa, Riggs, Dancu, & Rothbaum, 1993). The SEM at any time point would be 4.35 (SEM=10.54√1–0.83). Thus, the standard error of difference (SED) between two assessments would equal 6.15 points on the PDS (SED=√2(SEM)^2^). As reported in other studies (Devilly & Foa, 2001; Doane et al., 2010; Jun et al., 2013), a reliable change between two assessments of this scale would be a change of at least seven points. Therefore, we defined a change of seven points as cutoff score for a sudden change. A cutoff of seven points in the PDS indicates 13.7% of the total range (see also Jun et al., 2013), which is thus comparable to the standard provided by this study.
2. According the second criterion, sudden changes had to represent at least 25% of the score in the pre-gain/loss session (|score _*N+1*_ – score _*N*_| ≥0.25 * score _*N*_).
3. The third criterion required a significantly higher/lower mean score of the three sessions before the improvement/exacerbation than the mean scores of the three sessions after the gain/loss. However, we also included gains/losses when only two pre-change and post-change scores were available. A two-sample *t*-test with a 5% significance level was conducted to assess for significant differences. The following critical *t* values were used: *t*(4)>2.78, *p*=0.05; *t*(3)>3.19, *p*=0.05 (Lutz et al., 2013).

#### Implications of sudden changes

The frequency of sudden changes between treatment and waiting group was compared using Fisher's exact test and Mann-Whitney *U* Test (Hypotheses 1 & 2). To examine whether sudden gains are significantly more likely following sessions including exposure than during sessions without exposure, the frequency of sudden changes after exposure sessions was compared to the frequency of sudden changes after non-exposure sessions using paired *t* tests for dependent samples (Hypothesis 3). Two-sided *t* tests with absence/presence of sudden gains/losses as independent variables and PDS and CAPS as outcome variables were conducted to evaluate the influence of sudden gains/losses on treatment outcome (Hypothesis 4).

Logistic regression analyses examined the association between various patient characteristics with the presence of sudden changes during treatment. Presence/absence of sudden changes were treated as the dependent variables. Severity of PTSD symptoms at pre-treatment, number of diagnosis at pre-treatment, number of borderline-criteria at pre-treatment, NSSI pre-treatment and during treatment and level of depressive symptoms at pre-treatment were entered as predictor variables into the model.

All analyses were carried out using SPSS Version 22.

## Results

### Frequency of sudden gains and sudden losses

In total, 1,776 weekly PDS assessments were analysed to identify sudden changes across all participants and over the assessment period of 24 weeks. To clarify the time when sudden changes occur, we report sudden changes in two steps. We report the total number of sudden changes first. Then we report sudden changes for the treatment period and the follow-up period in the DBT-PTSD group which equals the first and the last 12 weeks of assessment in the TAU-WL.

In the DBT-PTSD group, sudden losses were observed in *n=*2 participants (5.6%), each reporting one instance of sudden loss. One sudden loss occurred during treatment, the other sudden loss was observed during the follow-up period. The symptom exacerbation per sudden loss was 11 points during the treatment period (21.6% of the total score) and seven points during the follow-up period (13.7% of the total score). Both instances of sudden losses did not result in a symptom worsening relative to the pre-treatment assessment.

In the TAU-WL group, sudden losses were observed in *n=*4 participants (10.5%) with one participant reporting two instances of sudden losses. During the first 12 weeks of waiting (equals the treatment phase in the DBT-PTSD group), three sudden losses in *n*=3 participants occurred. Two sudden losses in *n*=2 participants were observed during the last 12 weeks of waiting (follow-up period in the DBT-PTSD group). In one of these four participants, the symptom worsening during the waiting period resulted in a reliable worsening at the 24-week assessment relative to the pre-assessment. The symptom exacerbation per sudden loss was on average 12.50 points (SD=5.60, range 7–20; 24.5% of the total score).

The occurrence of sudden losses over the complete assessment period (24 weeks) did not differ significantly between DBT-PTSD and TAU-WL groups (Mann-Whitney *U* Test, *p=*0.42); neither did the number of participants reporting sudden losses (Fisher's exact test, two-sided, *p =* 0.68).

In the DBT-PTSD group, *n*=9 patients (25%) reported at least one sudden gain (*n=*8 during the 12-week treatment phase; *n*=1 during the follow-up). Most individuals in this subgroup had one instance of a sudden gain (*n=*6). Two participants had two instances of sudden gains. On average, the symptom reduction per sudden gain was 10.88 points (SD=2.17, range 9–15; 21.3% of the total score).

In the TAU-WL group, *n=*2 (5.3%) patients reported sudden gains during their waiting period with both reporting two instances of sudden gains. During the first 12 weeks of waiting (equals the treatment phase in the DBT-PTSD group), three sudden gains in *n=*2 participants occurred with one participant having two sudden gains. One sudden gain in *n=*1 participant was observed during the last 12 weeks of waiting (equivalent to the follow-up period in the DBT-PTSD group). On average, symptom reduction per sudden gain was 19.50 points (SD=3.70, range 15–24; 38.2% of the total score).

**Fig. 2:**
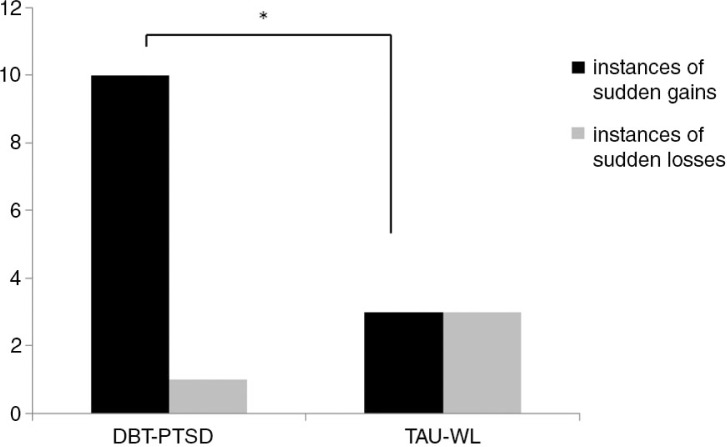
Sudden changes in the DBT-PTSD group during the 12 weeks treatment phase versus. TAU-WL during the first 12 weeks waiting period. *Fisher's exact test, two-tailed, *p*=0.04.

In line with Hypothesis 2, significantly more sudden gains were observed in the DBT-PTSD group than in TAU-WL group over the complete assessment period (Mann-Whitney *U* Test, *p=*0.03). The occurrence of sudden gains during the treatment period (first 12 weeks of assessment) also differed significantly between the groups (Mann-Whitney *U* Test, *p=*0.04; see Fig. 2). Significantly more participants reported sudden gains in the DBT-PTSD group than in the TAU-WL (Fisher's exact test, two-sided, *p=*0.02).

### Session content and sudden gains in the treatment group

**Fig. 3:**
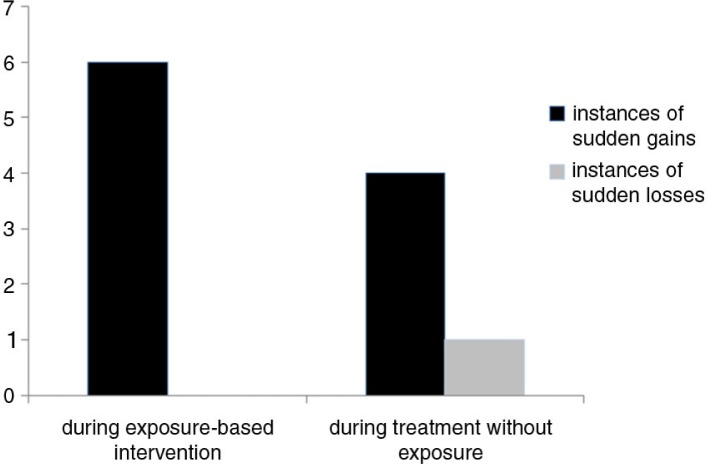
Number of sudden changes in the DBT-PTSD group during treatment.

Sudden gains occurred in different treatment phases. Six instances of sudden gains occurred during weeks with exposure interventions. Exposure-based interventions took place in *M=*4.06, SD=2.19 treatment weeks. The other four instances of sudden gains were reported in treatment weeks where no exposure was delivered (see Fig. 3). In average *M=*8.38, SD=2.13 treatment weeks did not include exposure interventions.

In order to compare the occurrence of sudden gains following exposure versus non-exposure interventions (Hypothesis 3), two indices were computed: 1) number of sudden gains in weeks with exposure interventions divided by the number of treatment weeks with exposure (sudden gains/number of weeks with exposure: *M*=0.04, SD = 0.09) and 2) number of sudden gains in weeks without exposure interventions divided by the number of treatment weeks without exposure (sudden gains/number of weeks without exposure: *M=*0.02; SD=0.05). The difference between the two indices did not reach significance, *t*(30)=1.60, *p=*0.12.

### Sudden changes and treatment outcome

Treatment outcome at post-treatment and follow-up assessments was measured with the CAPS and the PDS. *t-*Tests for each assessment time were conducted to test Hypothesis 4. As hypothesised, results revealed significantly better treatment outcomes at post-treatment and follow-up assessments in participants with sudden gains than participants without sudden gains for most assessment points (see Table 1).

**Table 1:** Treatment results: PTSD measurements in the DBT-PTSD group

	Participants with sudden gains (*n=*8)	Participants without sudden gains (*n=*28)	*t*(34)	*p*
CAPS pre-treatment	82.25 (16.02)	89.54 (13.51)	1.37	0.18
CAPS post-treatment	42.38 (24.22)	65.43 (25.61)	2.27	0.03
CAPS 6 weeks follow-up	44.50 (16.12)	61.18 (26.88)	1.67	0.11
CAPS 12 weeks follow-up	41.63 (18.74)	63.32 (23.66)	2.38	0.02
Cohen's *d* (pre–12 weeks follow-up)	2.33	1.36		
PDS pre-treatment	2.04 (0.41)	2.28 (0.44)	1.29	0.21
PDS post-treatment	1.19 (0.66)	1.73 (0.59)	2.23	0.03
PDS 6 weeks follow-up	1.18 (0.63)	1.63 (0.48)	2.21	0.03
PDS 12 weeks follow-up	1.18 (0.69)	1.63 (0.62)	1.76	0.09
Cohen's *d* (pre – 12 weeks follow-up)	1.52	1.21		

*Note*. Data are expressed as *M* (SD).

### Participant characteristics and sudden changes

As the number of sudden losses was very small, only the relationship between sudden gains and participant characteristics was examined. Participants with and without sudden gains did not differ significantly in age (Mann-Whitney *U* Test, *p=*0.07), PTSD symptom severity (PDS: *t*(34)=1.37, *p=*0.18; CAPS: *t*(34)=1.39, *p=*0.21), depressive symptoms *t*(34)=0.65, *p*=0.52) or in the number of Axis-1 diagnoses at pre-treatment (Mann-Whitney *U* Test, *p*=0.30). For more details see Table 2. In addition, NSSI at pre-treatment and during treatment did not predict sudden gains in logistic regression analyses, NSSI pre-treatment: *B*=0.36, *SE(B)*=1.00, *p*=0.72; NSSI during treatment: *B*=1.01, *SE ( B)*=0.91, *p*=0.27.

**Table 2:** Association between patient characteristics and sudden gains in the DBT-PTSD group

	Participants with sudden gains (*n*=8)	Participants without sudden gains (*n*=28)	*t*(34)	*p*
Age, years	29.75 (10.11)	36.96 (9.97)		0.07^a^
Number of axis I disorders—current	2.63 (0.74)	3.07 (1.05)		0.30^a^
Number of BPD criteria	4.75 (2.05)	4.07 (1.51)		0.40^a^
BDI (pre-treatment)	36 (8.5)	38.57 (10.14)	0.65	0.52
SCL-90-R (pre-treatment)	1.71 (0.55)	1.96 (0.69)	0.93	0.36
BSL (pre-treatment)	2.29 (0.56)	2.14 (0.73)	−0.54	0.60

*Note*. Data are expressed as *M* (SD).

a Mann-Whitney *U* Test.

## Discussion

The present study focused on the frequency of sudden changes and their association with treatment content and outcome in an RCT on DBT-PTSD versus TAU-WL. Clinicians often report concerns that exposure-based interventions in PTSD may lead to symptom worsening (Becker et al., 2004; Jaycox & Foa, 1996; Rosen et al., 2004). Beyond the measurement of symptom worsening from pre- to post-treatment, it appears important to investigate acute worsening during treatment and especially in relationship with exposure sessions. The concept of sudden gains was adopted to detect sudden losses. Although this might be a very conservative procedure to detect symptom worsening it seemed reasonable to use these criteria to detect acute worsening that goes beyond normal symptom fluctuation.

Sudden losses were extremely rare in the present study, with only one participant reporting a sudden loss during treatment and another one during follow-up. Importantly, these instances of symptom worsening did not follow exposure sessions and were only temporary as these participants did not show symptom worsening in the post- or follow-up assessment relative to pre-treatment. This finding is in line with the existing literature (Foa et al., 2002) and indicates that DBT-PTSD can safely be conducted, even in a highly chronic and impaired patient group. Whereas most of the earlier studies had severe comorbidity as an exclusion criterion, it was an inclusion criterion in this study. The current findings therefore extend earlier results to a population with more severe and comorbid symptomatology. Sudden losses occurred in less participants in the DBT-PTSD condition (*n*=2) compared to the waitlist condition (*n*=4), although this difference did not reach statistical significance. This finding is at odds with the frequently expressed concern that symptom worsening follows exposure-based interventions. However, the results have to be interpreted with caution since our results are based on a small sample size. The absence of sudden losses defined in a specific way does not prove a non-existence of any form of symptom worsening following exposure sessions. Future research should use and directly compare multiple criteria of symptom worsening. Two characteristics of the treatment programme may have influenced our results. First, DBT-PTSD is a phase-based programme, whereby participants are taught specific skills to control dysfunctional behaviour in the first phase. These skills may have helped participants to counter any symptom-worsening effects of exposure-based interventions in the second phase. Future research is needed to investigate whether the same results can be found without prior skills training. Second, DBT-PTSD was delivered as a residential programme and it remains to be tested whether findings generalize to an outpatient setting.

The current study not only focused on sudden losses, but also investigated sudden gains during DBT-PTSD. Results showed that sudden gains were not specific for the treatment group as they also occurred in the TAU-WL. However, in line with the hypotheses the number of sudden gains was significantly higher in the DBT-PTSD than in the TAU-WL group. In comparison to earlier studies reporting sudden gains in approximately 40–50% of participants receiving PTSD treatment (e.g., Kelly et al., 2009; Doane et al., 2010), the frequency of sudden gains was somewhat lower in the present study (25%). One reason for this difference could be the chronicity of the population, as all participants suffered from chronic PTSD after CSA. To our knowledge, no other research paper reports on sudden gains in PTSD following CSA, thus we lack knowledge about the typical symptom course during treatment in this specific group.

Importantly, however, sudden gains indicated better treatment outcome at the post-treatment assessment, which is in line with earlier research. This effect remained stable up to the 12 weeks follow-up assessment when looking at the CAPS ratings (but note that the parallel analysis using the PDS did not reveal statistically significant results at 12 weeks follow-up anymore). It is important to note the small statistical power for these analyses because of small numbers of sudden gains. Thus, these results have to be taken with caution and need to be replicated.

From a theoretical as well as clinical point of view, it is interesting to investigate whether sudden gains are more frequent following some interventions than others. Sixty percent of sudden gains occurred in treatment weeks just after exposure was delivered, the remaining 40% of sudden gains were observed during the first treatment phase with a focus on psycho-education, skills training, and cognitive interventions. The rates of sudden gains did not differ significantly between treatment phases, which might be due to the small sample size and the relatively low number of sudden gains. However, referring back to frequently described concerns regarding the safety of exposure-based treatments, it is important to emphasize the overall pattern of findings in that participants receiving exposure sessions did not show any sudden losses in response to these sessions, whereas the opposite phenomenon of sudden gains was observed in a substantial number of participants following exposure sessions. We also tested whether sudden gains could be predicted by patient characteristics. Consistent with other findings (Kelly et al., 2009; Doane et al., 2010), initial symptom severity and comorbidity were not predictive of sudden changes at all. Thus, sudden changes might better be explained by underlying change mechanisms, e.g., cognitive shifts or reduction in avoidance strategies.

The current study shows a number of strengths. This includes the use of a severely affected sample of CSA survivors as well as the test of associations between sudden changes and preceding interventions. Additionally, sudden symptom changes were examined not only during the treatment phase, but also during the follow-up period and in the waiting list condition.

On the other hand, some limitations are noteworthy. First, as the sample size and the number of sudden changes were relatively small, the statistical power was reduced for a number of analyses. Second, using the PDS for the weekly assessment includes some problems. As reported by Priebe et al. (2013), the PDS might not capture the whole range of symptoms. Thus, symptom changes in both directions—losses and gains—might be more often experienced by patients than our data suggest. Third, the current findings appear most informative in terms of the frequency of sudden changes and their association with treatment outcome. However, the results remain relatively silent on predictors of these symptom changes. Most importantly, the underlying mechanisms leading to sudden gains or losses are unclear. It is conceivable that either cognitive changes or new experiences through exposure interventions may precede sudden changes, but this remains to be tested empirically. In future studies, possible mechanisms of change should therefore be assessed in a systematic way. Finally, the generalizability of the current findings to an outpatient setting remains to be tested.

## Conflict of interest and funding

The study was funded by the German Research Foundation (DFG; STE1818/2-1).
